# Advanced Glycation End Products Enhance Murine Monocyte Proliferation in Bone Marrow and Prime Them into an Inflammatory Phenotype through MAPK Signaling

**DOI:** 10.1155/2018/2527406

**Published:** 2018-03-22

**Authors:** Xian Jin, Liang Liu, Yaping Zhang, Yin Xiang, Guizhi Yin, Yi Lu, Ludong Shi, Jian Dong, Chengxing Shen

**Affiliations:** ^1^Department of Cardiology, Shanghai Jiao Tong University Affiliated Sixth People's Hospital, Shanghai, China; ^2^Department of Cardiology, Xin Hua Hospital, Shanghai Jiao Tong University School of Medicine, Shanghai, China; ^3^Department of Cardiology, Minhang Hospital, Fudan University, Shanghai, China

## Abstract

**Objective:**

Increased monocytes, particularly the inflammatory subset, are associated with accelerated atherosclerosis in diabetes through thus far incompletely defined mechanisms. The present study tested the hypothesis that advanced glycation end products (AGEs) promote bone marrow monocytes to proliferate and drive them into an inflammatory phenotype.

**Methods and Results:**

In vivo, AGEs (25 mg/kg i.p. for 7 days) increased proportions of CD115^+^ monocytes and the inflammatory subset, the CD115^+^Ly6C^high^ cells, in murine bone marrow (flow cytometry analysis (FCM)), and enhanced gene expression of proinflammatory cytokines (IL-1*β* and TNF-*α*) but only slightly upregulated mRNA expression of anti-inflammatory cytokine (IL-10) (real-time PCR) in monocytes. In vitro, when the monocytes were treated with different dosages of AGEs (50, 150, and 300 *μ*g/mL), we found that proliferation (CCK8) but not apoptosis (FCM) of the monocytes was induced; the mRNA expressions of proinflammatory cytokines (IL-1*β* and TNF-*α*) and GM-CSF were upregulated in a dose-dependent manner while mRNA levels of IL-10 and M-CSF were changed much less in monocytes (real-time PCR). Furthermore, AGEs (300 *μ*g/mL) significantly enhanced the expression of Ki67 in monocytes (immunofluorescence staining (IF)), and this dose of AGEs markedly increased secretion of GM-CSF but not that of M-CSF (ELISA). For a pathway study, the monocytes were stimulated by 300 *μ*g/mL AGEs for different periods of time (0, 15, 30, and 120 min) and the activation of the MAPK pathway was tested (FCM); the results showed the p38 and ERK pathways were activated but not JNK signaling. Pretreatment with an inhibitor of p38 (SB203580) or ERK (U0126) attenuated AGE-induced gene expression of proinflammatory cytokines and GM-CSF (real-time PCR), as well as reversing AGE-induced Ki67 expression (IF).

**Conclusions:**

AGEs promote bone marrow monocytes to proliferate and drive them into an inflammatory phenotype through p38 and ERK activation.

## 1. Introduction

Monocytes and macrophages play vital roles in the formation and progression of atherosclerosis. Increased proportions of circulating monocytes, especially the inflammatory type, might be associated with accelerated atherosclerosis in diabetes which have been documented in diabetic Akita mice [1] and in STZ-induced diabetic mice [2, 3]. Nagareddy et al.'s work [2] demonstrated that high glucose drives bone marrow myelopoiesis and promotes diabetes-associated atherogenesis. However, the fact that achieving normoglycemia in diabetic mice failed to restore the counts of monocytes to normal level hinted that pathogenic factors other than hyperglycemia might also play roles in monocytosis. Advanced glycation end products (AGEs), a heterogeneous group of toxic molecules formed from proteins and sugar residues by irreversible nonenzymatic reactions in a hyperglycemic milieu, are also important pathogenic factors which stimulate inflammation and enhance atherogenesis in diabetes [4]. Previously, studies have demonstrated that AGEs promote mature macrophage growth [5] and enhance the cells differentiating into the classically activated phenotype [6]. It remains unknown if and through which mechanisms AGEs promote CD115^+^ monocytes to proliferate in the bone marrow and drive them into a proinflammatory phenotype before they differentiate into macrophages, which is the subject of the present study in mice.

## 2. Results

### 2.1. AGEs Increase Proportions of Bone Marrow CD115^+^ Monocytes and Their Inflammatory Subset

**Figure 1:**
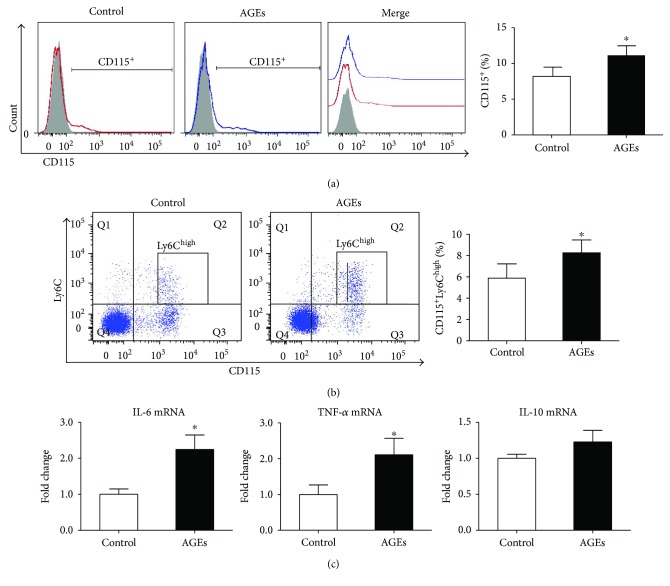
AGEs increased proportions of CD115^+^ monocytes and their inflammatory subset in the bone marrow. Mice were divided into control (*n* = 11) and AGE (*n* = 11) groups. The AGE group received AGEs (25 mg/kg) via intraperitoneal injection once a day for 7 consecutive days, while the control group received BSA. On the 8th day, 5 mice in each group were sacrificed and bone marrow mononuclear cells were isolated for surface marker analysis by flow cytometry. The other 6 mice in each group also were sacrificed, and bone marrow CD115^+^ monocytes were sorted for real-time PCR analysis. (a) Proportions of CD115^+^ monocytes in the bone marrow. Bar graphs represent the results (mean ± SD) of five independent experiments. (b) Proportions of CD115^+^Ly6C^high^ inflammatory monocytes in the bone marrow. Bar graphs represent the results (mean ± SD) of five independent experiments. (c) mRNA expression of IL-1*β*, TNF-*α*, and IL-10 in CD115^+^ monocytes. Bar graphs represent the results (mean ± SD) of three independent experiments. ^∗^*P* < 0.05 compared to the control group.

To assess the effects of AGEs on proportions of CD115^+^ monocytes in bone marrow, 22 mice were randomly divided into control (*n* = 11) and AGE (*n* = 11) groups and treated via intraperitoneal injection once a day for 7 consecutive days with 25 mg/kg bovine serum albumin (BSA) or AGE-BSA, respectively. On the eighth day, five mice randomly selected from each group were sacrificed, and bone marrow mononuclear cells were isolated and subjected to flow cytometry analysis to assess proportions of CD115^+^ monocytes and the CD115^+^Ly6C^high^ inflammatory subset. The other 6 mice in each group were also sacrificed, and CD115^+^ bone marrow monocytes were sorted using a magnet microbead kit. RNA was extracted, and mRNA levels of proinflammatory (IL-1*β* and TNF-*α*) or anti-inflammatory (IL-10) cytokines were assessed by real-time PCR. The results showed that both the proportions of CD115^+^ monocytes and the CD115^+^Ly6C^high^ inflammatory subset were higher in the AGE group (Figures 1(a) and 1(b)). AGEs also increased gene expression of proinflammatory cytokines but did not affect mRNA levels of anti-inflammatory cytokine in bone marrow monocytes (Figure 1(c)). Therefore, the results hinted that AGEs might promote proliferation of monocytes in the bone marrow and enhance their differentiation into a proinflammatory phenotype.

### 2.2. AGEs at Concentrations up to 300 *μ*g/mL Do Not Increase Apoptosis of Monocytes but Promote Their Proliferation In Vitro

**Figure 2:**
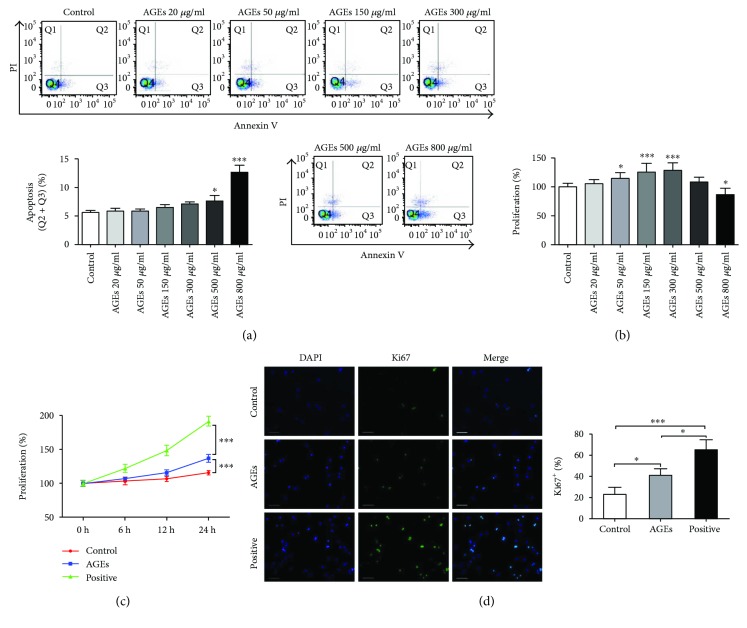
AGEs at concentrations up to 300 *μ*g/mL did not increase monocyte apoptosis but promoted their proliferation in vitro. For (a) and (b), CD115^+^ monocytes were treated with different concentrations of AGEs (20, 50, 150, 300, 500, and 800 *μ*g/mL) for 24 h; BSA was used as the control. To keep the cells alive, 15 ng/mL M-CSF was added to each group. Flow cytometry analysis was then performed for apoptosis evaluation and CCK8 assay for cell proliferation analysis. (a) Effects of different concentrations of AGEs on monocyte apoptosis. (b) Effects of different concentrations of AGEs on monocyte proliferation. Bar graphs represent the results (mean ± SD) of three independent experiments. ^∗^*P* < 0.05 and ^∗∗∗^*P* < 0.001 in between-group comparisons. For (c) and (d), CD115^+^ monocytes were divided into 3 groups (control, AGEs, and positive control). The AGE group was stimulated with 300 *μ*g/mL AGEs, the control group was incubated with the same concentration of BSA, and the positive control group was treated with a standard concentration of M-CSF (50 ng/mL). The control and AGE groups were treated with a low concentration of M-CSF (15 ng/mL) to keep the cells alive. After 24 h incubation, CCK8 assay and immunofluorescence staining for Ki67 were performed. (c) Effects of different treatments on monocyte proliferation. Bar graphs represent the results (mean ± SD) of three independent experiments. (d) Ki67 expression in monocytes after different stimulations. Bar graphs represent the results (mean ± SD) of four independent experiments. ^∗^*P* < 0.05 and ^∗∗∗^*P* < 0.001 in between-group comparisons.

To test the effects of AGEs on apoptosis or proliferation of bone marrow monocytes, CD115^+^ monocytes were treated with different concentrations of AGEs (20, 50, 150, 300, 500, and 800 *μ*g/mL) for 24 h; BSA was used as the control. To maintain cell viability, 15 ng/mL M-CSF was added to each group. Apoptosis was then assessed by flow cytometry analysis and cell viability and proliferation by CCK8 assay.  Figure 2(a) shows that AGEs at concentrations up to 300 *μ*g/mL did not increase monocyte apoptosis, while high concentrations of AGEs (≥500 *μ*g/mL) significantly induced monocyte apoptosis.  Figure 2(b) indicates that except at the highest concentration tested (800 *μ*g/mL), AGEs promoted cell proliferation. These results demonstrated that AGEs at concentrations up to 300 *μ*g/mL increased monocyte proliferation but did not significantly enhance apoptosis. Therefore, a dose of 300 *μ*g/mL was chosen for additional experiments (CCK8 assay and immunofluorescence staining for Ki67) to confirm AGEs' proliferative effects on bone marrow monocytes. CD115^+^ monocytes were divided into 3 groups (control, AGEs, and positive control). The AGE group was stimulated with 300 *μ*g/mL AGEs, the control group was incubated with the same concentration of BSA, and the positive control group was treated with a standard concentration of M-CSF (50 ng/mL). The control and AGE groups were treated with a low concentration of M-CSF (15 ng/mL) to keep the cells alive. For CCK8 assay, the cells were incubated for 6, 12, and 24 h. For immunofluorescence staining, the cells were incubated for 24 h. After incubation, CCK8 assay and immunofluorescence staining for Ki67 were performed.  Figure 2(c) shows that the standard concentration of M-CSF markedly promoted monocyte proliferation and that AGEs also significantly increased monocyte proliferation.  Figure 2(d) demonstrates that expression of Ki67, a marker of cell proliferation, was highest in the positive control group and lowest in the control group and that AGEs significantly increased Ki67 expression when compared with the control group.

### 2.3. AGEs Enhanced Expression of Proinflammatory Cytokines and GM-CSF in Monocytes In Vitro

**Figure 3:**
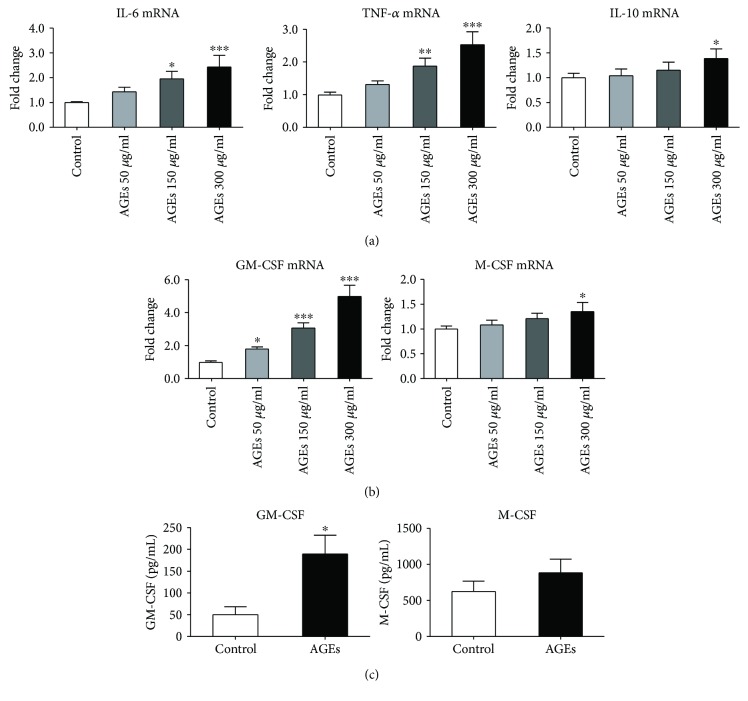
AGEs enhanced expression of proinflammatory cytokines and GM-CSF in monocytes in vitro. For (a) and (b), CD115^+^ monocytes were treated with different concentrations of AGEs (50, 150, and 300 *μ*g/mL) for 24 h; BSA was used as the control. Cells were then harvested, and RNA was extracted for real-time PCR tests. (a) Effects of AGEs on mRNA expression of proinflammatory cytokines (IL-1*β* and TNF-*α*) and anti-inflammatory cytokine (IL-10). (b) Effects of AGEs on gene expression of GM-CSF and M-CSF. Bar graphs represent the results (mean ± SD) of four independent experiments. ^∗^*P* < 0.05, ^∗∗^*P* < 0.01, and ^∗∗∗^*P* < 0.001 compared to the control group. For (c), the monocytes were treated with or without AGEs (300 *μ*g/mL) for 48 h; BSA was used as the control. The supernatant fluid was collected for ELISA detection. (c) Effect of AGEs on GM-CSF and M-CSF secretion by bone marrow monocytes. Bar graphs represent the results (mean ± SD) of three independent experiments. ^∗^*P* < 0.05 and ^∗∗^*P* < 0.01 compared to the control group.

Based on in vivo results, in vitro experiments were then performed to further substantiate AGEs' proinflammatory effects on bone marrow monocytes. CD115^+^ monocytes were treated with different concentrations of AGEs (50, 150, and 300 *μ*g/mL) for 24 h; BSA was used as the control. Next, cells were harvested and RNA was extracted. Real-time PCR analysis showed that AGEs significantly increased the mRNA expression of proinflammatory cytokines, IL-1*β* and TNF-*α*, in a dose-dependent manner, while the mRNA level of IL-10, an anti-inflammatory cytokine, slightly upregulated only when the highest concentration of AGEs was used (Figure 3(a)). Thus, the results underscored that AGEs drive bone marrow monocytes into a proinflammatory phenotype.

Experiments were then conducted to reveal the mechanisms underlying the effects of AGEs on monocyte proliferation. Due to GM-CSF and M-CSF being well-known colony-stimulating factors involved in monocyte proliferation and differentiation, levels of mRNA and proteins of GM-CSF as well as of M-CSF were assessed by real-time PCR and ELISA tests. For gene expression tests, monocytes were treated with different concentrations of AGEs (50, 150, and 300 *μ*g/mL) for 24 h, while BSA was used as the control; then, RNA was extracted for real-time PCR analysis. For protein measurement, monocytes were pretreated with or without AGEs (300 *μ*g/mL) for 48 h, and the supernatant fluid was collected for ELISA analysis.  Figure 3(b) shows that AGEs upregulated mRNA levels of GM-CSF in a dose-dependent manner while only slightly increasing mRNA levels of M-CSF.  Figure 3(c) confirms that AGEs markedly enhanced monocytes to secrete GM-CSF while only numerically but not significantly increasing M-CSF secretion. Therefore, the results indicated that AGEs promoted monocyte proliferation in the bone marrow at least partly by enhancing GM-CSF expression.

### 2.4. MAPK Pathway Was Involved in AGE-Induced Monocyte Proliferation and Proinflammatory Activation

**Figure 4:**
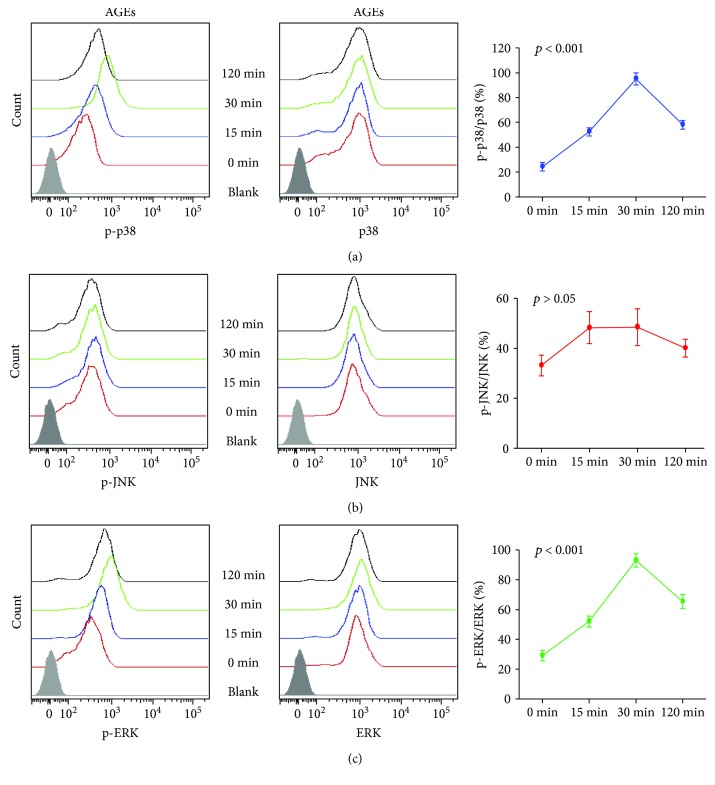
AGEs activated p38 and ERK pathways. Bone marrow monocytes were stimulated with AGEs (300 *μ*g/mL) for different time periods (0, 15, 30, and 120 min), and cells were then harvested for flow cytometry analysis. Levels of MAPK proteins (p38, ERK, and JNK) and their phosphorylated form (p-p38, p-ERK, and p-JNK) were measured as MFI (mean fluorescence intensity) by flow cytometry at different time points after AGE stimulation. The degree of pathway activation was assessed based on the ratio of the phosphorylated form to total protein. (a) AGEs activated the p38 pathway. (b) AGE did not activate the JNK pathway. (c) AGEs activated the ERK pathway. Each point represents the results (mean ± SD) of three independent experiments.

Seeing that the MAPK pathway has been reported to participate in macrophage proinflammatory activation [7, 8] and GM-CSF expression in various cells [9, 10], we investigated the possible involvement of the MAPK pathway in AGEs inducing monocyte proliferation and proinflammatory activation. First, the effects of AGEs on MAPK activation were assessed in bone marrow CD115^+^ monocytes. Monocytes were stimulated with AGEs (300 *μ*g/mL) for different periods of time (0, 15, 30, and 120 min), and cells were harvested for flow cytometry analysis. Levels of MAPK proteins (p38, ERK, and JNK) and their phosphorylated form (p-p38, p-ERK, and p-JNK) were measured as mean fluorescence intensity (MFI) by flow cytometry in different time points after AGE stimulation. The results showed that the p-p38/p38 and p-ERK/ERK ratios were very low before AGE stimulation (0 min) but sharply increased after AGE stimulation, peaked at 30 min, and decreased thereafter (Figures 4(a) and 4(b)). However, the ratio of p-JNK/JNK did not change markedly over time (Figure 4(c)). The latter finding indicated that AGEs activated the p38 and ERK pathways, but not JNK, in bone marrow CD115^+^ monocytes.

**Figure 5:**
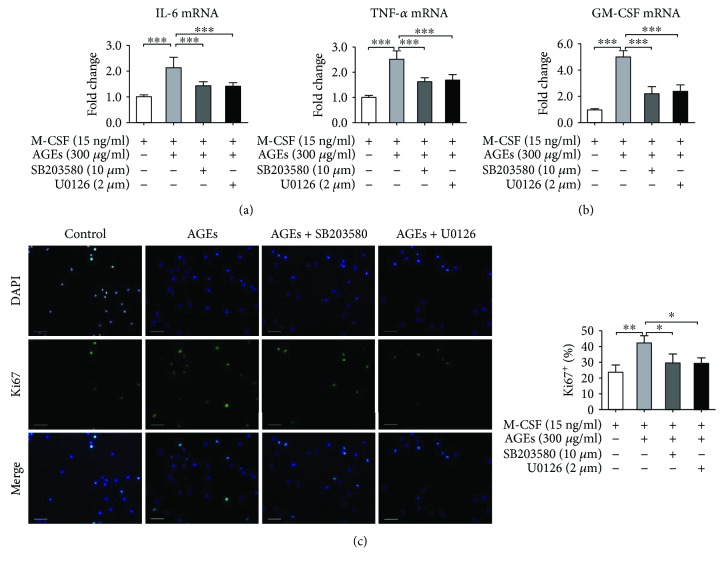
Block of the p38 or ERK pathway attenuated AGE-induced monocyte proliferation and proinflammatory activation. Cells were divided into four groups (control, AGEs, AGEs + SB203580, and AGEs + U0126). The control group was treated with BSA and the AGE group with AGEs (300 *μ*g/mL), while the next two groups were pretreated with SB203580 (specific inhibitor of p38) or U0126 (specific inhibitor of ERK) for 60 min and then treated with AGEs (300 *μ*g/mL) during a 24 h incubation. For (a) and (b), the 4 groups of cells were harvested and RNA was extracted for real-time PCR tests. (a) Block of p38 or ERK pretreatment attenuated AGE-induced proinflammatory cytokine (IL-1*β* and TNF-*α*) expression. (b) Block of p38 or ERK pretreatment attenuated AGE-induced mRNA expression of GM-CSF. For (c), cells were prepared for IF staining for Ki67. (c) Block of p38 or ERK pretreatment abated AGE-induced Ki67 expression in monocytes. Bar graphs represent the results (mean ± SD) of four independent experiments. ^∗^*P* < 0.05, ^∗∗^*P* < 0.01, and ^∗∗∗^*P* < 0.001, in between-group comparisons.

Next, expression levels of proinflammatory cytokines (IL-1*β* and TNF-*α*) and GM-CSF were assessed to confirm whether they are dependent on p38 and ERK signaling. Cells were divided into four groups (control, AGEs, AGEs + SB203580, and AGEs + U0126). The control group was treated with BSA and the AGE group with AGEs (300 *μ*g/mL), while the other two groups were pretreated with SB203580 (specific inhibitor of p38) or U0126 (specific inhibitor of ERK) for 60 min and then with AGEs (300 *μ*g/mL). After 24 h incubation, the 4 groups of cells were harvested and RNA was extracted; levels of mRNA of proinflammatory cytokines (IL-1*β* and TNF-*α*) and GM-CSF were then measured. Figures 5(a) and 5(b) show that both SB203580 and U0126 pretreatments abated the effect of AGEs on proinflammatory cytokine promotion and GM-CSF expression enhancement. In addition, IF staining for Ki67 was performed to confirm whether the proliferation of bone marrow monocytes was dependent on MAPK signaling or not.  Figure 5(c) demonstrates that pretreatment with SB203580 or U0126 significantly attenuated AGE promotion of Ki67 expression in monocytes. Therefore, the results indicated that p38 and ERK pathways were implicated at least partially in the proinflammatory response and AGE enhancement of monocyte proliferation.

## 3. Discussion

The present study provided two novel insights into the effects of advanced glycation end products on bone marrow monocytes, the precursors of the macrophages. Firstly, AGEs promoted the proliferation of CD115^+^ monocytes in the bone marrow and favored a shift to a proinflammatory phenotype. Secondly, the effects of AGEs on bone marrow monocytes were at least partly dependent on p38 and ERK signaling.

Previous studies have shown increased proportions of circulating monocytes, especially inflammatory monocytes, in both type 1 and 2 diabetic mouse models [2, 3, 11]. Hyperglycemia has been verified to be associated with monocytosis in diabetes. However, the hypoglycemic therapy failed to reverse the monocytes to normal level indicating that hyperglycemia was not the only causal factor. Although the effects of AGEs on monocyte proliferation had not been documented, other studies had reported their proliferative effects on other cells [12–14]. To our knowledge, the present study is the first to report AGE-induced proliferation of monocytes in the bone marrow, which could partly underlie monocytosis in diabetes.

GM-CSF and M-CSF are considered the primary cytokines stimulating monocytes to proliferate and differentiate [15], and various factors promote monocyte/macrophage proliferation via GM-CSF production [2, 16]. The present study replicated the finding of AGE-enhanced GM-CSF expression in monocytes. Although M-CSF gene expression was upregulated by the highest concentration of AGEs, ELISA tests showed that AGEs failed to significantly enhance monocyte secretion of M-CSF. Therefore, GM-CSF production appears mainly responsible for AGEs inducing proliferation of monocytes. However, the mechanisms for AGE-induced monocytosis are probably far from simple. Firstly, in this study, we only studied the effects of AGEs on the monocyte autosecretion profile but did not assess the possible impact of AGEs on the production of GM-CSF by other cells in the bone marrow. Secondly, AGEs were also found to promote the monocyte to express IL-1*β*, which had been shown to drive proliferation of myeloid cells in the bone marrow [11] and to promote GM-CSF production [17, 18]. Thus, mechanisms of AGE-induced monocyte proliferation beyond promotion of GM-CSF secretion warrant further elucidation.

Although the proinflammatory roles of AGEs in mature monocytes/macrophages have been well documented [3, 19–21], the effects of AGEs on monocytes in the bone marrow, the relatively immature monocytes, remain poorly understood. In vivo experiments in the present study showed that AGEs increased the proportions of CD115^+^Ly6C^high^ monocytes, the inflammatory subset, in the bone marrow, and in vitro experiments showed that AGEs predominantly upregulated the expression of proinflammatory cytokines in these cells. The latter results indicated that AGEs not only promoted bone marrow monocyte proliferation but also enhanced their shift into an inflammatory phenotype. Hence, the present study provided the novel insight that AGEs can prime the immature monocytes into a proinflammatory phenotype before they mature.

Though there had been no reports on the pathway underlying AGE-induced GM-CSF expression, several known pathways including MAPK, NF-kappaB, and PI3K/AKT pathways were found to be involved in AGE proinflammatory effects on monocytes/macrophages [6, 8, 20, 21]. Moreover, MAPK signaling has been reported to partake in GM-CSF production in a variety of cells other than monocytes and macrophages [9, 10, 22]. Therefore, these previous studies indicated that the MAPK pathway might be responsible for AGEs inducing expression of GM-CSF and proinflammatory cytokines in the bone marrow monocytes. The present study showed that AGEs activated p38 and ERK MAPK pathways, which significantly attenuated the increased gene expression of proinflammatory cytokines and GM-CSF when inhibited. Furthermore, p38 or ERK signaling blockade reversed the AGE-mediated increase in ki67 expression. Thus, p38 signaling and ERK signaling were here shown to be involved in AGE-induced bone marrow monocyte proliferation and proinflammatory activation.

Advanced glycation end products are well known as critical pathogenic factors in diabetes with the ability to enhance atherosclerosis, through mechanisms that remained to be defined. The present study demonstrated that AGEs induced monocytes in the bone marrow to proliferate and enhance their shift into a proinflammatory phenotype at least partly through p38 and ERK signaling. These findings provide insight into the mechanisms underlying increased proportions of circulating monocytes and their inflammatory subset in diabetes, with AGEs probably acting as a major mediator linking enhanced atherosclerosis and diabetes. Strategies to reverse or block the effects of AGEs on bone marrow monocyte proliferation and proinflammatory activation might conduce to slowing the progression of diabetes-associated atherosclerosis.

## 4. Methods and Materials

### 4.1. Mice

Male C57BL/6 mice (8 weeks old) were purchased from Slac Laboratory Animal Co. Ltd. (Shanghai, China). The mice were housed under specific pathogen-free conditions with controlled temperature (22–25°C) and 12 h light/dark cycles; they were given standard chow and water ad libitum. All animal experiments were approved by the Ethics Committee of Xinhua Hospital affiliated to Shanghai Jiao Tong University School of Medicine (approval number XHEC-F-2016-016).

For in vitro experiments, bone marrow CD115^+^ monocytes were isolated by cell sorting.

For in vivo experiments, mice (*n* = 22) were randomly assigned to control (*n* = 11) and AGE (*n* = 11) groups. Mice in the AGE group received 25 mg/kg AGEs (purified AGE-BSA, Anyan-bio Technology, Shanghai, China, catalog number AY4710P) via peritoneal injection once a day for 7 consecutive days, while the control group received the same volume of BSA. On the eighth day, five mice randomly selected from each group were sacrificed and bone marrow mononuclear cells were isolated and characterized by flow cytometry analysis. The other 6 mice of each group were sacrificed on the 8th day, and bone marrow monocytes were sorted by a magnet microbead kit and RNA was extracted to perform real-time PCR tests.

### 4.2. Preparation of Bone Marrow Mononuclear Cells (BM-MNCs) and Sorting and Culture of CD115^+^ Monocytes

Mice were anesthetized with pentobarbital (50 mg/kg, i.p.) to minimize suffering and killed by cervical dislocation without recovery from anesthesia. The tibias and femurs were removed, bone ends were cut off, and the bone marrow was flushed with buffer solution (PBS supplemented with 1% fetal bovine serum (FBS, Gibco, Australia, catalog number 10099-141)). A single-cell suspension was prepared by filtering the cells through a 40 *μ*m strainer (BD Falcon, catalog number 352340). The cell suspension was centrifuged at 1500 rpm for 5 min and the cell pellet collected.

BM-MNCs were isolated by density gradient centrifugation with Histopaque® 1077 (Sigma-Aldrich, USA, catalog number 10771) at 400*g* for 30 min at 18°C.

Bone marrow CD115^+^ monocytes were sorted by a CD115 microbead kit (Miltenyi Biotec, Bergisch Gladbach, Germany, catalog number 130-096-354) according to the manufacturer's instructions. The purity of the sorted cells (>90%) was assessed by flow cytometry with an anti-CD115 APC antibody (eBioscience, California, USA, catalog number 17-1152). For in vivo experiments, the CD115^+^ cells were used to extract RNA for real-time PCR tests. For in vitro experiments, bone marrow monocytes were cultured at 37°C with 5% CO_2_ in Dulbecco's modified Eagle's medium (DMEM) High Glucose (HyClone, Beijing, China, catalog number SH30022.01B) supplemented with 10% FBS, 2 mM L-glutamine (Sigma-Aldrich, Missouri, USA, catalog number 59202C), 100 U/mL penicillin, and 100 *μ*g/mL streptomycin (Beyotime, Jiangsu, China, catalog number C0222). To keep the cells alive, 15 ng/mL of M-CSF (PeproTech, New Jersey, USA, catalog number 315-02) was added into the culture medium.

### 4.3. Flow Cytometry Analysis

For analysis of surface markers of monocytes in vivo, BM-MNCs were prepared at a density of 10^6^/mL in flow buffer. Then, 100 *μ*L cell suspension was incubated with antibodies. Anti-mouse CD115 APC was used to identify monocytes, and anti-mouse Ly6C percp-cy5.5 (eBioscience, California, USA, catalog number 45-5932) for their inflammatory subset.

For analysis of apoptosis of CD115^+^ monocytes in vitro, bone marrow CD115^+^ monocytes (1 × 10^6^/mL) were seeded in 6 cm plates, cultured overnight, and then incubated with different concentrations (20, 50, 150, 300, 500, and 800 *μ*g/mL) of AGEs (purified AGE-BSA, Anyan-bio Technology, Shanghai, China, catalog number AY4710P) for 24 h; BSA (Sigma-Aldrich, Missouri, USA, catalog number A1933) was used as the control vehicle. To keep the cells alive, 15 ng/mL of M-CSF was added to each well. After 24 h incubation, cells were harvested, and the Annexin V FITC Assay kit (BD Biosciences, California, USA, catalog number 556420) was used to detect monocyte apoptosis.

For MAPK pathway research, flow cytometry analysis was performed as previously described [23]. Monocytes (1 × 10^6^/mL) were seeded in 6 cm plates, cultured overnight, and then stimulated with AGEs (300 *μ*g/mL) for different time intervals (0, 15, 30, and 120 min) followed by harvesting for flow cytometry analysis. Levels of MAPK proteins (p38, ERK, and JNK) and their phosphorylated form (p-p38, p-ERK, and p-JNK, resp.) were measured as mean fluorescence intensity (MFI) by flow cytometry at different time points after AGE stimulation. Anti-phospho-p38 antibody, anti-p38 antibody (catalog numbers 4511S and 8690S), anti-phospho-ERK antibody, and anti-ERK antibody (catalog numbers 9101S and 4695S) were purchased from Cell Signaling Technology (Massachusetts, USA). Anti-phospho-JNK antibody and anti-JNK antibody (catalog numbers ab124956 and ab208035) were purchased from Abcam (Cambridge, UK). The secondary antibody conjugated with Alexa Fluor 488 was purchased from Beyotime (Jiangsu, China, catalog number A0423).

The results were acquired by a BD Canto II flow cytometer (Becton-Dickinson Biosciences) and analyzed with FlowJo (Tree Star Inc., Ashland, OR, USA).

### 4.4. Cell Proliferation Assay

CCK8 assay was performed for cell proliferation detection. Bone marrow monocytes (5 × 10^4^/well) were seeded in 96-well plates and cultured overnight before stimulation. To keep the cells alive, 15 ng/mL M-CSF was added to each plate.

To assess the effect of different concentrations of AGEs on cell proliferation, cells were incubated with 20, 50, 100, 150, 300, 500, and 800 *μ*g/mL of AGEs for 24 h; BSA was used as the control vehicle.

To assess the effect of AGE stimulation duration on cellular proliferation, cells were treated with 300 *μ*g/mL AGEs for various time periods (0, 6, 12, and 24 h). BSA was used as the control vehicle; cells treated with 50 ng/mL M-CSF were used as the positive control.

All cells were cultured at 37°C with 5% CO_2_. After incubation, 10 *μ*L CCK-8 solution (DOJINDO, Kumamoto, Japan, catalog number CK-04) was added into each well followed by another 4 h incubation. Then, the optical density (OD) values were measured at 450 nm using a microplate reader (BioTek, USA). The cell proliferation rate of treated cells was calculated relative to the control group.

### 4.5. Immunofluorescence Staining

Bone marrow CD115^+^ monocytes (1 × 10^6^/mL) were seeded in 6-well plates and cultured overnight. Based on experimental aims, monocytes were treated with different reagents for about 24 h. Cells were then washed twice with PBS and fixed in 4% paraformaldehyde for 10 min, then washed again with PBS. Next, cells were incubated for 1 h at room temperature in buffered normal goat serum to prevent nonspecific binding of antibodies. Next, they were incubated separately overnight with an antibody purchased from CST against Ki67 (dilution 1 : 400; Boston, USA, catalog number 9129T), followed by incubation with Alexa Fluor 488 goat anti-rabbit IgG (dilution 1 : 500; Beyotime, Jiangsu, China, catalog number A0423) for 1 h at 37°C. Thereafter, cells were washed with PBS. 40,6-Diamidino-2-phenylindole (DAPI) was used to stain the cell nuclei (blue) at a concentration of 1.43 *μ*M (catalog number D8417; Sigma). Photomicrographs were taken with a DMI3000B camera (Leica, Germany).

### 4.6. Real-Time PCR Analysis

For in vivo experiments, RNA was extracted directly from sorted bone marrow CD115^+^ monocytes without culture in vitro.

For in vitro experiments, bone marrow CD115^+^ monocytes (1 × 10^6^/mL) were seeded in 6 cm plates and cultured overnight. Based on experimental aims, cells were stimulated with different reagents. After incubation for about 24 h, cells were harvested. Total RNA was extracted from monocytes using TRIzol reagent (catalog number 9109; Takara, Liaoning, China), and 1 *μ*g of total RNA was reverse transcribed to cDNA using the PrimeScript RT Master Mix kit (catalog number RR036A; Takara). Real-time PCR array analysis was performed using the SYBR Premix Ex Taq™ kit (catalog number RR420A; Takara) in a total volume of 20 *μ*L with 0.4 *μ*L of primers, 10 *μ*L of SYBR Green and 0.4 *μ*L of Rox Dye II, and 2 *μ*L of cDNA. Standard PCR conditions included 95°C for 30 s, followed by 40 cycles of 95°C for 5 s and 60°C for 34 s, with a final dissociation stage, and samples were run on an ABI 7500 detector (Applied Biosystems, Foster City, CA, USA). Amounts of target genes were determined and normalized to that of GAPDH cDNA (catalog number B661304; Sangon Biotech, Shanghai, China). The sequences of the primers (synthesized by Sangon Biotech) for the target genes were as follows: Fwd 5′-CTCACAAGCAGAGCACAAGC-3′ and Rev 5′-TCCAGCCCATACTTTAGGAAGA-3′ for IL-1*β*, Fwd 5′-GGTGCCTATGTCTCAGCCTC-3′ and Rev 5′-CCACTTGGTGGTTTGTGAGTG-3′ for TNF-*α*, Fwd 5′-TGCACTACCAAAGCCACAAG-3′ and Rev 5′-TGATCCTCATGCCAGTCAGT-3′ for IL-10, Fwd 5′-TGAGTCTGTCTTCCACCTGCT-3′ and Rev 5′-CCAATGTCTGAGGGTCTCG-3′ for M-CSF, and Fwd 5′-TGGTCTACAGCCTCTCAGCA-3′ and Rev 5′-GACGACTTCTACCTCTTCATTCAAC-3′ for GM-CSF.

### 4.7. Enzyme-Linked Immunospecific Assays (ELISA) for Cytokine Detection

After adjusting a single-cell suspension of monocytes to a density of 1 × 10^6^ cells/mL, 1 mL cell suspension was added into each well of 12-well plates and cultured overnight. On the next day, bone marrow monocytes were treated with BSA or AGEs (300 *μ*g/mL). After 48 h stimulation, the supernatant fluid was collected and levels of GM-CSF and M-CSF (Abcam, Cambridge, UK, catalog numbers ad201276 and ab199084) were determined using commercially available ELISA kits according to the manufacturer's instructions. To keep the cells alive for GM-CSF detection, 15 ng/mL M-CSF was added into the culture medium; to keep the cells alive for M-CSF detection, 5 ng/mL GM-CSF was added into the culture medium.

### 4.8. Statistical Analysis

All results are expressed as mean ± SD. Student's *t*-test was used for between-group comparisons. One-way analysis of variance was used to assess the effects of one factor among multiple groups, and post hoc testing was performed using Tukey's test. Two-way analysis of variance was used to assess the effects of two factors among multiple groups, and post hoc testing was performed using the Bonferroni test. Analysis was performed using SPSS, version 19.0 (IBM Corp., Armonk, NY, USA). Two-tailed significance levels are reported. *P* < 0.05 was considered statistically significant.
